# AK2‐Deficient Mice Recapitulate Impaired Lymphopoiesis of Reticular Dysgenesis Patients, but Also Lack Erythropoiesis

**DOI:** 10.1002/eji.202451466

**Published:** 2025-07-14

**Authors:** Rebekka Waldmann, Franziska Werner, Alpaslan Tasdogan, Felix Immanuel Maier, Ursula Kohlhofer, Irene Gonzalez‐Menendez, Leticia Quintanilla de Fend, Amrit Kaur Puarr, Ruth Maree Arkell, Anselm Enders, Manfred Hoenig, Hubert Schrezenmeier, Hans Joerg Fehling, Klaus Schwarz, Ulrich Pannicke

**Affiliations:** ^1^ Institute For Transfusion Medicine Ulm University Ulm Germany; ^2^ Institute For Clinical Transfusion Medicine and Immunogenetics Ulm German Red Cross Blood Transfusion Service Baden‐Wuerttemberg‐Hessen and University Hospital Ulm Ulm Germany; ^3^ Institute of Immunology University Hospital Ulm Germany; ^4^ Department of Dermatology University Hospital Essen and German Cancer Consortium Essen Germany; ^5^ Division of Immunology and Infectious Disease, John Curtin School of Medical Research The Australian National University Canberra ACT Australia; ^6^ Department of Pediatrics Universal Medical Center Ulm Ulm Germany; ^7^ Institute of Pathology and Neuropathology and Comprehensive Cancer Center Tuebingen Eberhard‐Karls‐University Tuebingen Germany; ^8^ Cluster of Excellence iFIT (EXC2180) “Image‐Guided and Functionally Instructed Tumor Therapies” Eberhard‐Karls‐University Tuebingen Germany; ^9^ Maternal and Foetal Precision Health, John Curtin School of Medical Research The Australian National University Canberra ACT Australia

**Keywords:** AK2‐deficient mouse model, impaired lymphopoiesis, reticular dysgenesis, severe anaemia

## Abstract

Reticular dysgenesis (RD) is a rare genetic disorder caused by mutations in the adenylate kinase 2 (*AK2*) gene. It is characterized by a T^−^B^−^ severe combined immunodeficiency, agranulocytosis, and sensorineural deafness. We established and characterized a haematopoiesis‐specific conditional *Ak2*‐knockout mouse model to provide a model system to study the molecular pathophysiology of RD. As expected from the human phenotype of RD, haematopoiesis‐specific AK2‐deficient embryos had a small, atrophic thymus consisting mainly of epithelial cells. No recognizable T‐cell component was observed, but B‐cell lineage precursor cells were present in the foetal liver. The effects of AK2 deficiency on myelopoiesis were less severe in mice than in humans. The absolute numbers of monocytes, macrophages, granulocytes and megakaryocytes in foetal liver as well as colony‐forming precursors were not reduced. In contrast to humans, haematopoiesis‐specific *Ak2*‐knockout mice exhibit embryonic lethality between E13 and E15 due to severe anaemia caused by an early block in definitive erythropoiesis. Murine erythroid progenitors mainly express AK2 and only low levels of functionally related kinases, which are unable to compensate for AK2 deficiency, in contrast to human erythroid progenitors.

## Graphical abstract

Ak2 deficiency leads to impaired development of T and B cells in mice, corresponding to impaired lymphopoiesis of reticular dysgenesis (AK2 deficiency) in humans. In contrast to the human pathophysiology, Ak2 deficiency is lethal in mice due to severe anaemia, and murine myelopoiesis is less affected.

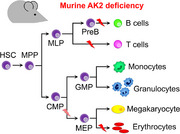

**Abbreviations**
AK2: adenylate kinase 2
BFU‐E: erythroid burst‐forming units
CFU‐G: granulocyte colony‐forming units
CFU‐GEMM: granulocyte, erythroid, macrophage, and megakaryocyte colony‐forming units
CFU‐GM: granulocyte and macrophage colony‐forming units
CFU‐M: macrophage colony‐forming units
CFU‐pre‐B: pre‐B colony‐forming units
iCre: codon‐improved Cre recombinase
LSK: lineage negative, Sca‐1^+^ and c‐Kit^+^
RD: reticular dysgenesis

## 1 Introduction

Reticular dysgenesis (RD) is a rare, drastic form of severe combined immunodeficiency. It is caused by inherited mutations of adenylate kinase 2 (*AK2*) [1, 2] and is characterized by impaired T‐ and B‐cell development, agranulocytosis and sensorineural deafness [3, 4, 5]. In RD patients, the thymus and secondary lymphoid organs are hypoplastic. T cells and granulocytes are either lacking or the numbers are far below normal ranges. However, numbers of B and NK cells can either be reduced or occasionally be within the normal range. Myeloid differentiation is blocked at the pro‐myelocytic stage. Almost half of the RD patients show decreased haemoglobin levels and/or reduced numbers of platelets at birth [1, 2, 6, 7].

The early onset of severe combined immunodeficiencies, the rarity of patients, and the life‐threatening condition of patients, in combination with early hematopoietic stem cell transplantation, limit the availability of patients’ materials for biomedical research. Animal models are an established alternative to overcome these obstacles and may recapitulate the pathology of human diseases. Mouse models have been successfully established for most genes defective in severe combined immunodeficiency patients [8, 9, 10, 11, 12, 13, 14, 15, 16, 17, 18, 19, 20, 21, 22, 23, 24, 25, 26, 27] and mostly mimic the human phenotype. Other murine models, for example, LIG4‐ [11, 12] or ADA‐deficiency models [8, 18, 26, 27] develop a more severe phenotype in mice when compared with humans.

In this study, a conditional, haematopoiesis‐specific *Ak2* knockout mouse line was established and characterized to provide a model system for further studies on the molecular biology and pathophysiology of RD. The haematopoiesis‐specific murine *Ak2* defect perfectly reproduces T and partially granulocyte deficiency of human RD patients. Yet, in stark contrast to RD patients, mice show an impaired erythrocyte development and die in utero.

## 2 Results

### 2.1 Haematopoiesis‐Specific Biallelic *Ak2*‐Knockouts Survive Past E13.5 but Not Past E18.5 Allowing Analysis of Foetal Haematopoiesis

To fundamentally investigate the pathophysiological basis of the very rare AK2 deficiency in humans, we generated Ak2‐deficient mouse models. To overcome embryonic lethality of a constitutive *Ak2* knockout (Supporting Information Results), the *iCre* recombinase transgene [28] under the control of the haematopoiesis‐specific *Vav* promoter [29] was used for a haematopoiesis‐specific, conditional *Ak2*‐knockout model. Heterozygous *Ak2^+/−^* mice with a vav‐iCre transgene were mated with homozygous *Ak2^fl/fl^* mice to generate *Cre^−^Ak2^fl/^* ^+^, *Cre^−^Ak2^fl/−^*, *Cre^+^Ak2^fl/+^*, and *Cre^+^Ak2^fl/−^* mice, with the latter representing haematopoietic *Ak2*‐knockouts.

The frequency of conditional knockout embryos was normal until E13.5 (Table S2). There was no difference in size between E13.5 animals with different genotypes. The liver of conditional knockout embryos appeared macroscopically paler than the liver of controls (Figure S3A). All organs exhibited normal development, but histologically, the foetal livers of conditional knockout embryos showed decreased numbers of erythroblastic islands (Figure S3B). Of note, the absolute number of foetal liver cells was strongly reduced in *Cre^+^Ak2^fl/−^* mice (Figure S3C).

At E18.5, knockout embryos were detectable at a significantly reduced frequency of 10% (Table S2). The knockout embryos at E18.5 were smaller in size and were macroscopically easily identifiable by their pale appearance. Among live‐born mice, no conditional knockouts were detected (Table S2).

RD patients exhibit a profound lymphopoietic and myelopoietic developmental block, with about 40% being anaemic at birth [7]. E13.5 and E18.5 conditional knockout embryos were therefore analyzed for their hematopoietic stem and progenitor populations as well as for leukopoietic and erythropoietic capacity.

### 2.2 In the Haematopoietic Stem and Progenitor Cell Compartment, Megakaryocyte‐erythroid Progenitors (MEP) Are Affected by a Haematopoiesis‐specific *Ak2*‐knockout

At E13.5, the foetal liver is the main organ of murine haematopoiesis; therefore, the haematopoietic capacity of E13.5 foetal livers of *Cre^+^Ak2^fl/‐^* mice was analyzed at the stem and progenitor cell level.

**FIGURE 1:**
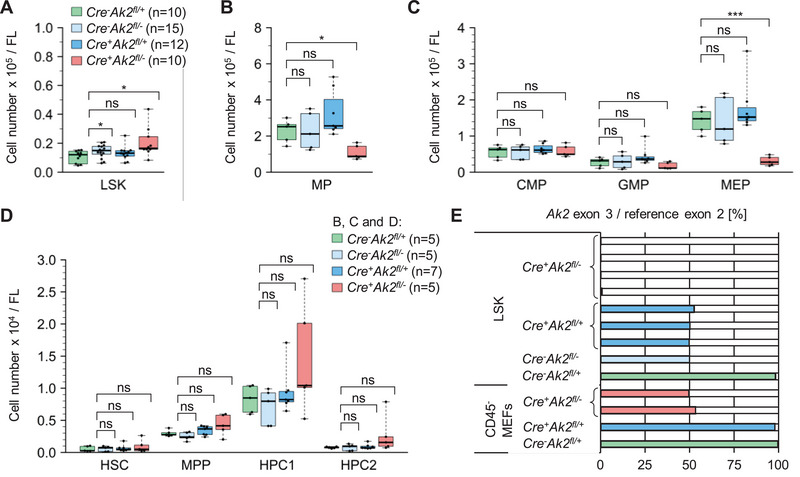
Flow cytometry analyses of haematopoietic stem and progenitor cells from foetal livers (FL) of conditional Ak2‐knockout embryos at E13.5. Numbers of (A) LSK cells, (B) multipotent progenitors (MP) (C) common myeloid progenitors (CMP), granulocyte‐macrophage progenitors (GMP) and megakaryocyte‐erythrocyte progenitors (MEP) and (D) hematopoietic stem cells (HSC), multipotent hematopoietic progenitors (MPP), and hematopoietic progenitor cells (HPC1 + HPC2). (E) Digital PCR analyses of the haematopoiesis‐specific deletion of the Ak2 exon 3 compared with the reference exon 2 in sorted LSK cells and CD45^−^ murine embryonic fibroblasts (MEFs) of the indicated genotypes. Each bar represents one single sample.

The surface markers c‐Kit and Sca‐1 were used to identify multipotent lineage negative (CD4^−^, Gr‐1^−^, Ter‐119^−^, CD8a^−^, B220^−^, CD3e^−^, CD5^−^), Sca‐1^+^ and c‐Kit^+^ (LSK) cells (Figure s4A). The number of E13.5 LSK cells per foetal liver of *Cre^−^Ak2^fl/−^* and *Cre^+^Ak2^fl/−^* LSK cells were statistically slightly increased (Figure 1A).

The lineage^−^Sca‐1^+^c‐Kit^−^ myeloid progenitors (MP) were subdivided according to their CD34 and CD16/32 (Fcγ receptor) expression [30] into common myeloid progenitors (CMP), granulocyte‐macrophage progenitors (GMP) and megakaryocyte‐erythrocyte progenitors (MEP) (Figure S4A). The absolute number of MP per foetal liver was reduced in *Cre^+^Ak2^fl/−^* mice (Figure 1B) in line with a markedly reduced number of viable MEP (Figure 1C; Figure S4B,C). The number of viable cells in the CMP and GMP cell subsets were unaffected by the *Ak2* genotype (Figure 1C).

LSK cells were subdivided into haematopoietic stem cells (HSC), multipotent haematopoietic progenitors (MPP), and haematopoietic progenitor cells (HPC1 + HPC2) according to their expression of the signalling lymphocyte activation molecule (SLAM) markers CD150 and CD48 [31, 32] (Figure S4A). Absolute numbers of HSC, MPP, HPC1 and HPC2 per foetal liver showed no statistically significant difference in conditional knockouts (Figure 1D).

The specificity and efficiency of the Cre‐mediated recombination of the floxed *Ak2* alleles, resulting in a deletion of *Ak2* exons 3 and 4, were determined by digital PCR in sorted CD45 negative murine embryonic fibroblast (MEF) controls and sorted LSK cells of foetal livers at E13.5. While no recombination was detected in fibroblasts, floxed alleles were almost completely (<1%) deleted in all Cre^+^ haematopoietic cells (Figure 1E).

### 2.3 Myeloid Progenitors of E13.5 *Cre^+^Ak2^fl/−^* Mice Are Numerically Not Reduced

AK2‐deficient patients show a generalized leukopenia that affects both the lymphoid and myeloid cell compartments [7].

**FIGURE 2:**
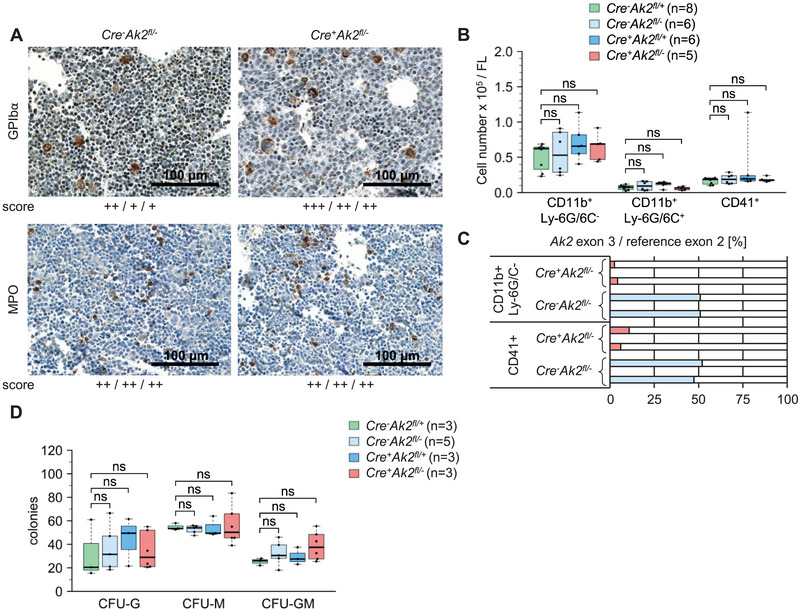
Myeloid development in conditional *Ak2*‐knockout embryos. (A) GPIbα (megakaryocytes) and MPO (myeloblasts) staining of foetal liver sections at E13.5. The score represents a semiquantitative evaluation of three samples as defined in Figure S5. (B) Number of CD11b^+^Gr‐1^−^ monocytes/macrophages, CD11b^+^Gr‐1^+^ granulocytes and CD41^+^ megakaryocytes per foetal liver (E13.5). (C) Digital PCR analyses of the hematopoiesis‐specific deletion of the *Ak2* exon 3 compared with the reference exon 2 in sorted CD11b^+^Gr‐1^−^ and CD41^+^ cells. Each bar represents one single sample. (D) Representative numbers of CFU‐G, CFU‐M and CFU‐GM colonies. CFU per 3 × 10^4^ viable foetal liver cells of embryos at E13.5.

The effect of AK2 deficiency on myeloid cells in the mouse model was investigated by immunohistochemical staining of the foetal liver, flow cytometry analyses and colony assays. Immunohistochemically, E13.5 *Cre^+^Ak2^fl/−^* mice appeared to exhibit slightly more megakaryocytes (GPIbα) and normal amounts of myeloblasts (MPO) in the foetal liver when compared with control embryos (Figure 2A). Calculation of the absolute cell numbers of myeloid populations per foetal liver according to their CD41, CD11b, and Ly‐6G/6C expression showed no significant differences between samples of different genotypes (Figure 2B; Figure S6).

Validation of the knockout efficiency by digital PCR in the CD11b^+^Ly‐6G/6C^−^ and CD41^+^ cell populations of *Cre^+^Ak2^fl/‐^* mice revealed 3% to 11% non‐deleted *Ak2* alleles (Figure 2C). Therefore, an enrichment of intact *Ak2* alleles was shown in myeloid populations of conditional knockout embryos when compared with LSK cells (< 1%) (Figure 1E). Due to the limited number of CD11b^+^Ly‐6G/6C^+^ cells per foetal liver, digital PCR analyses were not quantifiable.

In addition, myeloid differentiation potential was analyzed in numeric colony assays. The numbers of granulocyte colony‐forming units (CFU‐G), macrophage colony‐forming units (CFU‐M) and granulocyte and macrophage colony‐forming units (CFU‐GM) of *Cre^+^Ak2^fl/−^* mice were not significantly different compared with other genotypes (Figure 2D).

### 2.4 AK2 influences Murine B‐ and T‐cell Development

As lymphoid cells can be severely reduced in reticular dysgenesis patients [7], the T‐ and B‐cell developmental capacities were analyzed in the conditional Ak2‐knockout mice.

**FIGURE 3:**
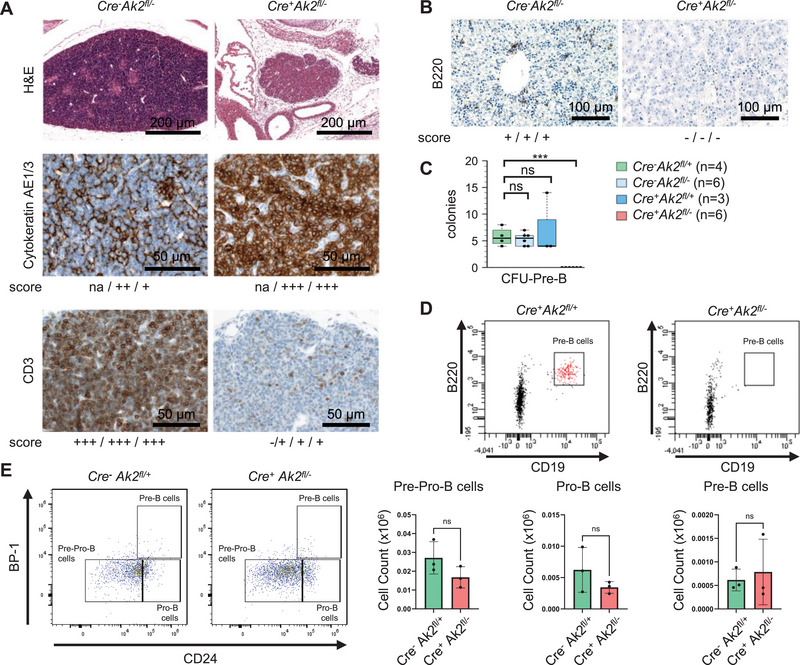
Lymphoid development in conditional *Ak2*‐knockout embryos. (A) Haematoxylin and eosin (H&E), AE1/3 (epithelial cells) and CD3 (T cells) staining of thymus sections at E18.5. (B) B220 (B cells) staining of foetal liver sections at E13.5. (A, B) The score represents a semiquantitative evaluation of three samples as defined in Figure S5. na: score not available. (C) Pre‐B colony‐forming potential of 1.5 × 10^5^ viable foetal liver cells from E13.5. (D) Representative examples of the flow cytometric analyses of the B220 and CD19 expression (pre‐B cells) of foetal liver cells after 7 days of the pre‐B‐cell colony assay. (E) Representative examples of the flow cytometric analyses of the B‐cell precursor fractions of foetal liver cells (*n* = 3 for each mouse strain) and total cell counts of populations A (pre‐pro‐B cells), B (pro‐B cells) and C (pre‐B cells) of Cre‐*Ak2^fl/+^* and Cre+*Ak2^fl/−^*, calculated by using live cell counts (Figure S7). Cells were pregated on time, single cells, live cells, and B220+CD43+.

Immunohistochemical analyses of E13.5 *Cre^+^Ak2^fl/‐^* embryonic thymi were not feasible due to the small size of the thymus, but E18.5 thymi were analyzed (Figure 3A). The thymus of AK2 expressing control embryos was well developed and filled with ample numbers of CD3‐positive thymocytes, as expected for a normal thymus. The thymus of *Cre^+^Ak2^fl/‐^* mice was smaller, atrophic and essentially without CD3^+^ thymocytes. Thymus epithelial cells were detectable. Cytokeratin AE1/3 staining showed evenly spread epithelial cells in the control thymus, while the thymus of E18.5 *Cre^+^Ak2^fl/−^* embryos was completely occupied by epithelial cells.

B220 immunohistochemistry of foetal livers at E13.5 revealed few positive cells in control and no B220‐positive cells in *Cre^+^Ak2^fl/‐^* samples (Figure 3B). *In vitro* B‐cell differentiation of *Cre^+^Ak2^fl/−^* cells was tested by colony assays (Figure 3C). Foetal liver cells of conditional knockout embryos at E13.5 showed no pre‐B colony‐forming units (CFU‐pre‐B). Only foetal liver cells of *Cre^−^Ak2^fl/+^*, *Cre^−^Ak2^fl/−^* and *Cre^+^Ak2^fl/+^* embryos were able to produce colonies containing B220^+^ and CD19^+^ cells (Figures 3C,D). Based on a flow cytometry staining and gating strategy of murine bone marrow, we analyzed B‐cell progenitors in foetal liver cells of *Cre^−^Ak2^fl/+^* and *Cre^+^Ak2^fl/−^* mice (Figure S7A). No significant differences were detected in living cell counts or the B220+CD43+ population (Figures S7B,C). The median B220 fluorescence was reduced in B220+CD43+ cells (Figure S7D). Moreover, FACS analyses of early B‐cell precursors in foetal liver at E13.5 showed no significant differences between *Cre^−^Ak2^fl/+^* and *Cre^+^Ak2^fl/−^* in the B220+CD43+ population for the cell counts in its fractions A, B, and C containing Pre‐Pro‐, Pro‐, and Pre‐B cells (Figure 3E).

### 2.5 Haematopoiesis‐Specific *Ak2* Deletion Leads to Embryonic Lethality Likely Due to Impaired Foetal Liver Erythropoiesis

Null mutations of AK2‐deficient patients are not lethal [1, 2], yet Ak2‐deficient murine embryos die preterm. To track the cause of murine embryonic lethality, the development of erythroid progenitors and mature erythrocytes in foetal livers was investigated by immunohistochemistry and flow cytometry (CD71 and Ter‐119) at E13.5.

**FIGURE 4:**
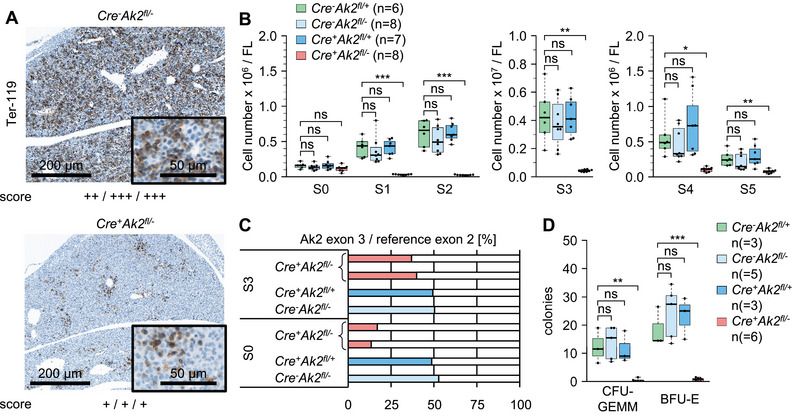
Erythroid development in conditional *Ak2*‐knockout embryos at E13.5. (A) Erythroid Ter‐119 staining of foetal liver sections. The score represents a semiquantitative evaluation of three samples as defined in Figure S5. (B) Number of erythroid progenitor cells per foetal liver. Viable cells were separated into six subsets (S0–S5) according to their Ter‐119 and CD71 expressions. (C) Digital PCR analyses of the haematopoiesis‐specific deletion of the *Ak2* exon 3 compared with the reference exon 2 in sorted foetal liver cells of the erythroid subsets S0 and S3. Each bar represents one single sample. (D) Numbers of CFU‐GEMM and BFU‐E per 3 × 10^4^ viable foetal liver cells after 12 days incubation time in semi‐solid methylcellulose‐based medium.

Erythropoietic activity was decreased in *Cre^+^Ak2^fl/−^* mice liver immunohistochemistry to only a few clusters of Ter‐119‐positive cells (Figure 4A). According to their CD71 and Ter‐119 expression, these erythroid foetal liver cells were subdivided into six maturation subsets named S0 to S5 by flow cytometry [33] (Figure S8A). A highly significant lack of foetal liver erythroid progenitors was detected in the subsets S1 to S5 of E13.5 *Cre^+^Ak2^fl/‐^* mice (Figure 4B). While absolute cell numbers of the subset S0 were similar in all genotypes (Figure 4B), the percentage of viable cells was reduced to approximately 20% in E13.5 *Cre^+^Ak2^fl/‐^* mice livers (Figure S8B).

Digital PCR analyses of sorted S0 and S3 cells revealed that the presence of Cre‐mediated recombination of the floxed *Ak2* allele was markedly reduced (Figure 4C) when compared with LSK cells (Figure 1C). Cells with intact *Ak2* alleles were enriched in the S0 and even more in the S3 subset. When compared with *Cre^−^Ak2^fl/−^* cells, 25–35% *Cre^+^Ak2^fl/−^* S0 cells and 75–80% S3 cells still carried a non‐deleted allele, hinting at the dependence of these subsets on AK2 function (Figure 4C).

Additionally, erythroid differentiation capability was assessed in ex vivo colony assays. In contrast to all other genotypes, the E13.5 *Cre^+^Ak2^fl/−^* foetal liver cells generated no granulocyte, erythroid, macrophage, and megakaryocyte colony‐forming units (CFU‐GEMM) and erythroid burst‐forming units (BFU‐E) (Figure 4D).

### 2.6 Differential Protein Expression of Adenylate and Creatine Kinases in Human and Murine Erythroid Development

**FIGURE 5:**
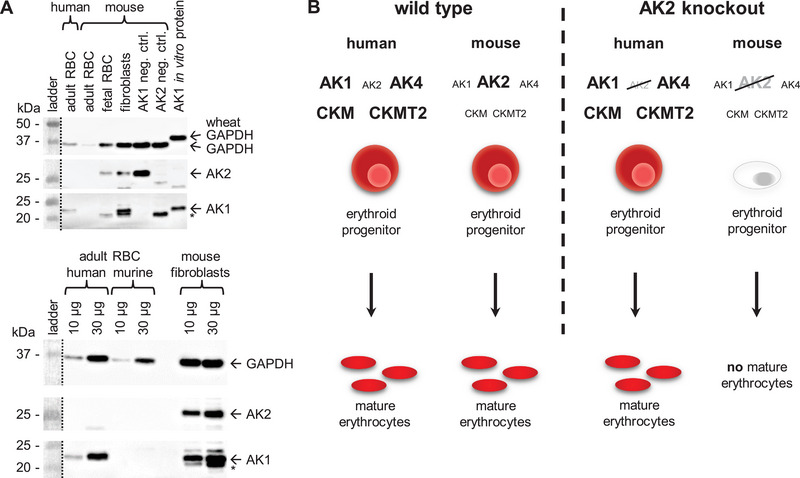
Differential expression of AK1 and other selected kinases during erythropoiesis: A hypothesis for the differing effects of an AK2 deficiency in human and mice. (A) Protein expressions of AK1 and AK2 in human and murine erythrocytes. Loading control: mammalian and wheat GAPDH, AK1 neg. ctrl.: BA/F3 cell line, AK2 neg. ctrl.: *Ak2* ^−/−^ murine ES cells. * Unspecific band (see Figure S10) (B) Schematic figure of the AK2‐independent human erythroid development compared with the AK2‐dependent murine erythroid development. Bold font: high protein expression, small font: no or low protein expression, grey font: knocked out gene. Dashed lines: pre‐stained protein standard ladders were documented separately to chemiluminescence detection.

Based on the differential AK2 dependence of human and murine erythropoiesis, western blot analyses of adenylate kinases were performed with protein lysates of human and murine adult erythrocytes and murine foetal erythrocytes. In adult erythrocytes of mice, neither AK1 nor AK2 protein expression was detected, whereas murine foetal erythrocytes express only AK2 protein. In contrast, human adult erythrocytes express AK1 (Figure 5A). An analysis of published mRNA sequencing data of normal human and murine haematopoietic cells, as provided by the BloodSpot database [34], revealed in support of our western blot data a differential expression of adenylate and creatine kinases in human and mice, which are known to compensate for AK2 [35] (Figure S9).

## 3 Discussion

In order to study its pathophysiology, a constitutive as well as a haematopoiesis‐specific Ak2‐knockout mouse model of the life‐threatening human congenital disorder RD were established.

When compared with human RD, these Ak2‐deficient strains only partially reflect the human situation.

In contrast to humans with complete AK2 deficiency, in both models, no live offspring were recorded. In the constitutive AK2‐deficient model, mice die shortly after nidation around E6.5 as reported [35, 36, 37]. Since first haematopoietic cells emerge at embryonic day 7 [38], this early mortality is most‐likely due to a non‐haematopoietic effect and was similarly seen in AK2 deficiency in inbred mice [35, 36, 37] and fruit flies [39, 40] but not in zebrafish [41] and humans [3, 7, 42]. In the haematopoiesis‐specific *Ak2*‐knockouts, the vast majority of progeny die past E13.5, yet prior to E18, most likely due to anaemia (discussed below). Thus, only the haematopoiesis‐specific *Ak2*‐knockout model yielded some information on stem cell and lympho‐/hematopoietic development.

As already shown in human RD patients [42], the absolute numbers and the viability of the haematopoietic stem and progenitor cell subsets, here, HSC, MPP, HPC1 and HPC2, were not affected by murine Ak2 deficiency. Furthermore, the absolute numbers of LSK cells per foetal liver were within the previously published range for LSK cells at E12.5–E15.5 [43, 44]. Verification of the efficiency and specificity of vav‐iCre‐mediated Ak2‐locus recombination yielded a recombination efficiency of ≥ 99% in LSK cells. No recombination was detected in non‐haematopoietic CD45^−^ MEFs. Therefore, AK2 function in mice, as in humans, seems not to be required for HSPC development and function.

Analogously, as in human AK2 deficiency [1, 2], T‐cell lymphopoiesis was influenced by Ak2 absence in mice. Murine conditional knockout embryos had a small, atrophic thymus consisting mainly of epithelial cells with CD45^+^ haematopoietic precursors but no recognizable T‐cell component.

An alternative approach to the use of mouse models of human diseases is to study the impact of pathogenic variants on cell development and/or function by the application of human organ cultures. In fact, an Artificial Thymic Organoid system based on a murine stromal cell line has already been used to test T‐cell differentiation from AK2‐deficient human haematopoietic stem and progenitor cells [45]. Compared with the culture of cells from healthy donors, only very few early haematopoietic progenitor cells were found, and almost no cells showed differentiation towards T cells, supporting our murine findings. This reflects very well the severe T‐cell lymphopenia in the known AK2‐deficient patients.

In FACS analyses of early B‐cell development of thawed foetal liver samples, *Cre^−^Ak2^fl/+^* and *Cre^+^Ak2^fl/−^* embryos (E13.5) showed comparable cell counts for the B220+CD43+ population and pre‐pro‐, pro‐ and pre‐B cell progenitors. These observations suggest that early B‐cell development still occurs in the foetal liver of *Ak2‐*deficient mice, as has been described in healthy human foetal liver [46]. This contrasts with the observed lack of B‐cell development in colony assays with murine AK2‐deficient cells, suggesting that the foetal liver may provide some signals essential for early B‐cell development or survival.

In immunohistochemistry of murine foetal livers of *Cre^−^Ak2^fl/−^* mice, the signal of B220 was rare and low and undetectable in AK2‐deficient *Cre^+^Ak2^fl/−^* mice. This may be explained by a reduced median B220 expression on foetal liver B‐cell progenitors of *Cre^+^Ak2^fl/−^* mice.

Agranulocytosis is a hallmark of human RD [4], which already manifests itself in clonogenic precursor cells as AK2‐knockdown progenitor cells have poor proliferative and survival capacities and are blocked in their differentiation towards granulocyte lineages [42]. In addition, RD patient granulopoiesis does not respond to granulocyte colony‐stimulating factor (G‐CSF) [42], explaining the granulocyte developmental block.

In contrast to human AK2‐deficiency, absolute numbers of monocytes/macrophages, granulocytes and megakaryocytes, as well as the respective clonogenic cells in mouse foetal liver, were unaffected when compared with wild‐type mice.

A significantly decreased fate decision of MEP towards the erythroid lineage and an early block in definitive erythropoiesis results in severe anaemia of conditional Ak2‐knockout embryos and likely leads to embryonic lethality of these mice between E13.5 and E18.5, similar to erythropoietin receptor‐deficient mice [33, 47]. Transition from S0 to S1 phase requires cell cycle S‐phase progression [33]. A negative effect of mitochondrial malfunction, caused by AK2 deficiency, on S‐phase progression was published for cardiomyocytes [48]. We, therefore, assume that AK2‐deficient cells of the S0 subset may not meet metabolic requirements at the cell cycle restriction point(s) and die, instead of entering S‐phase. Flow cytometry findings were confirmed by colony assays. No CFU‐GEMM and BFU‐E were detected among murine AK2‐deficient cells. An erythroid maturation defect caused by AK2 deficiency has also been described in the zebrafish model [43].

In contrast, human patients with RD are viable, and about half of them show haematological abnormalities affecting erythropoiesis [7]. We hypothesize that in light of the very low absolute numbers of human (outbred) RD patients reported some so far unknown genetic or epigenetic features with partial or complete compensation may seldom rescue human RD foeti from anaemic lethality. Future genomic analyses may help to resolve this conundrum.

To address a second mutually not exclusive hypothesis, why AK2 deficiency leads to different phenotypes in humans and mice, protein and RNA expression differences of kinases were assessed. Haematopoietic cells, which are negatively affected by an AK2 deficiency, express only AK2 and no or low levels of AK1, AK4, CKMT2 or CKM protein, while non‐affected fibroblasts and human erythrocytes additionally or exclusively express AK1, AK4, CKMT2 or CKM protein. These kinases are known to be able to substitute for AK2 [35, 48, 49]. We suggest that AK2 deficiency is directly causative of the erythroid defect in mice but spares human erythropoiesis due to these differences in kinase expression (summarized in Figure 5B). Human erythrocytes are hardly susceptible to an AK2 deficiency because erythroid progenitors express adenylate kinases like AK1 and AK4 and creatine kinases like CKMT2 and CKM. These enzymes are able to substitute for AK2 when the transition into lineage‐committed progenitors and proliferation requires more energy [50, 51, 52]. In contrast, the murine erythroid progenitors express mainly AK2 and only low levels of AK1, AK4, CKMT2 and CKM and are therefore not able to compensate for a loss of AK2 function in high‐energy demanding situations, when they mainly rely on efficient mitochondrial oxidative phosphorylation.

### 3.1 Data Limitations and Perspectives

There are weaknesses to this study: First, the study was performed in one inbred mouse strain; analyses of further breedings to other strains with distinct genetic backgrounds may help to detect variations in stem cell and lympho‐/hematopoietic development. Secondly, single‐cell RNA analyses of foetal liver and other tissues may help to define exact blocks in lineage trajectories. Thirdly, this study is purely descriptive, mostly due to the very low cell numbers in the respective compartments. A future refinement of the analytic armamentarium, especially in the area of single‐cell energy metabolism analyses, will help to address relevant mechanistic questions.

## 4 Materials and Methods

### 4.1 Mice

*Ak2^fl/fl^* mice were generated at TACONIC (New York/Cologne), by introducing loxP sites into intronic regions flanking exons 3 and 4 of the murine *Ak2* gene on a C57BL/6NTac background (Figure S1). *Ak2‐knockout* mice were generated by mating mice with one floxed and one wild‐type *Ak2* allele (*Ak2^fl/+^* mice) with *Cre* deleter mice [53] (B6.C‐Tg(CMV‐cre)1Cgn/J, Jackson Laboratories, Bar Harbor, USA, strain #: 006054). The obtained heterozygous *Ak2^+/−^* mice were mated to obtain a constitutive knockout. For the conditional *Ak2*‐knockout model, heterozygous *Ak2^+/−^* mice with a codon‐improved Cre recombinase (iCre) transgene under the control of the haematopoiesis‐specific *Vav* promoter (vav‐iCre [29]) were mated with homozygous *Ak2^fl/fl^* mice. Vav‐iCre mice were obtained from Jasper de Boer and Adam Williams (Department of Molecular Immunology, National Institute for Medical Research, London, GB) [29] and had been backcrossed onto a C57BL/6 background for at least 10 generations.

### 4.2 Flow Cytometry Analyses and Cell Sorting

Detailed information on cell processing, stainings, the antibodies used and the material sources for cell sorting are provided in the Supporting Information.

### 4.3 Histology

Organs were fixed in 4% formalin and paraffin‐embedded. For histology, 3–5 µm‐thick sections were cut and stained with haematoxylin and eosin (H&E). Immunohistochemistry was performed on an automated immunostainer (Ventana Medical Systems, Inc.) following the company's protocols for open procedures with slight modifications. The slides were stained with the antibodies anti‐GPIbα (emfret Analytics GmbH & Co. KG, Eibelstadt, Germany), anti‐MPO (anti‐myeoloperoxidase Ab‐1, Lab Vision UK, Ltd., Newmarket, Suffolk), anti‐Cytokeratin AE1/3 (Dako, Glostrup, Denmark), anti‐CD3 (Clone SP7, DCS Innovative Diagnostik‐Systeme GmbH u. Co. KG, Hamburg, Germany), anti‐B220 (Clone RA3‐6B2, BD Biosciences, Becton, Dickinson and Company, Franklin Lakes, New Jersey) and anti‐Ter119 (DakoCytomation, Glostrup, Denmark A/S). Appropriate positive and negative controls were used to confirm the adequacy of the staining. Images were acquired with an Axioskop 2 plus Zeiss microscope with a Jenoptik (Laser Optik System, Jena, Germany) ProgRes C10 plus camera and software. Final image preparation was performed with Adobe Photoshop CS6.

### 4.4 Colony Assays

Colony assays were performed in duplicate with E13.5 foetal liver cells. The MethoCult GF M3434 medium (Stemcell Technologies, #03434) was used following the manufacturer's instructions with 3 × 10^4^ foetal liver cells per 35 mm dish (Greiner bio‐one, cat. # 627102). Pre‐B cell colony assays were done with MethoCult M3630 medium (Stemcell Technologies, #03630) and 1.5 × 10^5^ foetal liver cells per 35 mm dish. Colonies were evaluated and counted by light microscopy (Nikon, inverted microscope Eclipse TE 300), and pre‐B‐cell colonies were additionally verified by flow cytometry.

### 4.5 Western Blot and Digital PCR Procedures

Specified in Supporting Information Methods.

### 4.6 Statistical Analyses

When appropriate, the variance compared with Cre^−^Ak2^fl/+^ mice was calculated by a two‐tailed Welch's *t*‐test. ns *p* > 0.05, *p ≤ 0.05, **p ≤ 0.01, ***p ≤ 0.001.

## Author Contributions

Rebekka Waldmann designed and realized the experiments, analyzed data, and wrote the manuscript. Franziska Werner, Alpaslan Tasdogan and Hans Joerg Fehling provided technical assistance and instructed for the isolation and FACS analyses of the murine foetal livers. Ursula Kohlhofer, Irene Gonzalez‐Menendez, and Leticia Quintanilla de Fend performed the histological stainings and analyses. Hubert Schrezenmeier provided funding and contributed to the study design. Felix Immanuel Maier, Anselm Enders, Manfred Hoenig, Klaus Schwarz, and Ulrich Pannicke designed experiments and analyzed data. Felix Immanuel Maier and Anselm Enders performed FACS of foetal liver cells. Amrit Kaur Puarr and Ruth Maree Arkell provided fresh foetal liver cells from WT animals. All authors edited the manuscript.

## Conflicts of Interest

The authors declare no conflicts of interest.

## Ethics Approval Statement for Animal Studies

All mice were maintained and bred at the animal facility of Ulm University in a specific pathogen‐free environment using individually ventilated cages under approved conditions of the animal facility and the regional administrative council with the project license z./o.168. Food and drinking water were available ad libitum during a 14/10 h day and night rhythm. The handling of all animals was in accordance with the local regulations of the animal welfare committee, and mice were sacrificed by carbon dioxide (gradual filling) according to the EU directive 2010/63/EU. All work with wildtype mice and embryos used for comparison with the frozen foetal liver samples of Ak2‐deficient mice was done under ethics protocol A2024/345 at the John Curtin School of Medical Research, Australian National University, Canberra, Australia.

## Supporting information




**Supporting Information file 1**: eji6021‐sup‐0001‐SuppMat.docx

## Data Availability

The data that support the findings of this study are available from the corresponding author upon reasonable request.
