# Supplementary material for: AK2‐Deficient Mice Recapitulate Impaired Lymphopoiesis of Reticular Dysgenesis Patients, but Also Lack Erythropoiesis
Source: Eur J Immunol. 2025 Jul 14;55(7):e51466. doi: 10.1002/eji.202451466 (PMC12257577; doi:10.1002/eji.202451466)
Supplement: Supplementary file 1 — Supporting Information file 1: eji6021‐sup‐0001‐SuppMat.docx [file EJI-55-e51466-s001.docx]

# Supporting information

1. Supplemental Figures 1 - 10
2. Supplemental Tables 1 - 4
3. Supplemental Methods
4. Supplemental Results
5. Supplemental References
6. Western Blot raw data

## Supplemental Figure 1


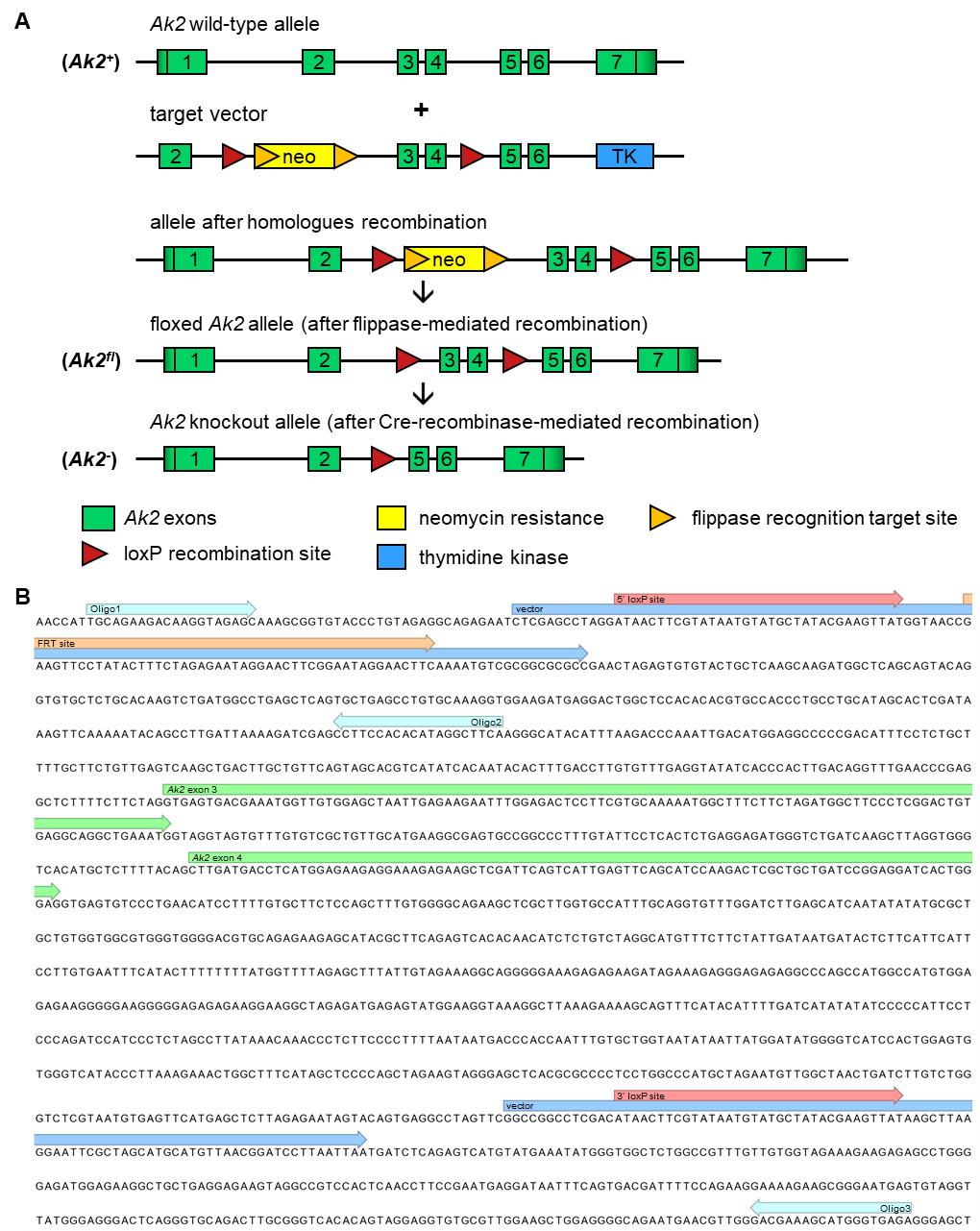


Supplemental Figure 1: Generation of floxed Ak2 (Ak2fl) and Ak2-knockout (Ak2-) alleles in mice after homologues recombination of a target vector with the wild-type allele in embryonic stem cells. (A)The cartoon depicts the generation of all *Ak2* alleles used in this study. (B) Genomic sequence of the floxed *Ak2* allele. Oligo 1 – 3 were used for genotyping as described in the supplemental methods section.

## Supplemental Figure 2


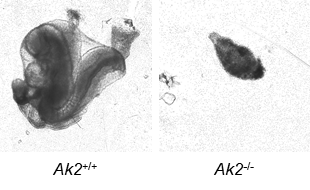


Supplemental Figure 2: *Ak2* deficiency in mice is lethal at Theiler stage 9 (around E7). *Ak2*-knockout embryos (*Ak2^-/-^*) isolated at E8.5 are characterized by growth retardation and their morphology corresponds to Theiler stage 9 [1] of E6.25–E7.25. Wild type (*Ak2^+/+^*) and heterozygous embryos are indistinguishable on gross inspection and correspond to the expected Theiler stage 12 of E7.5 to E8.75.

## Supplemental Figure 3


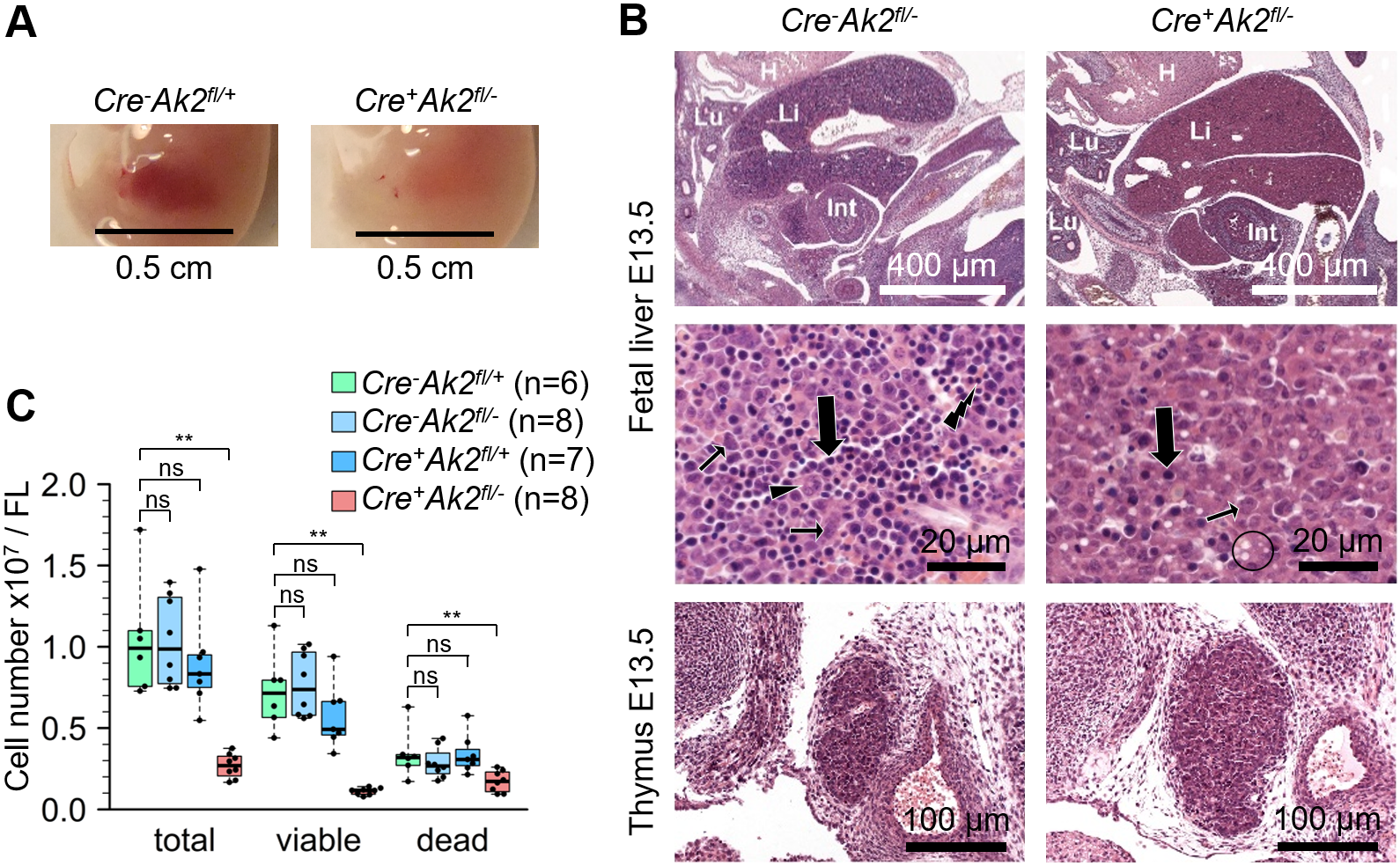


Supplemental Figure 3: Effects of a haematopoiesis-specific conditional *Ak2*-knockout
(*Cre^+^Ak2^fl/-^*) at E13.5. (A) Representative pictures of *Cre^-^Ak2^fl/+^* and haematopoiesis-specific *Cre^+^Ak2^fl/-^*knockout embryos. Throughout the study there were no differences at all between *Cre^-^Ak2^fl/+^* and *Cre^-^Ak2^fl/-^ or Cre^+^Ak2^fl/+^* embryos. (B) Hematoxylin and eosin staining of foetal liver and thymus sections. Li: liver, Lu: lung, Int: intestine, H: heart, small arrow: hepatoblasts, thick arrow: erythroblasts, lightning flash: nucleated erythrocytes, arrowhead: megakaryocytes, circle: intracytoplasmic inclusions. (C) Foetal liver cells were stained with SYTOX® Blue viability dye and the number of viable and dead cells was determined by flow cytometry. The variance compared to *Cre^-^Ak2^fl/+^* was calculated by two-tailed Welch’s t-test, ns p > 0.05, ** 0.01 ≥ p > 0.001.

## Supplemental Figure 4


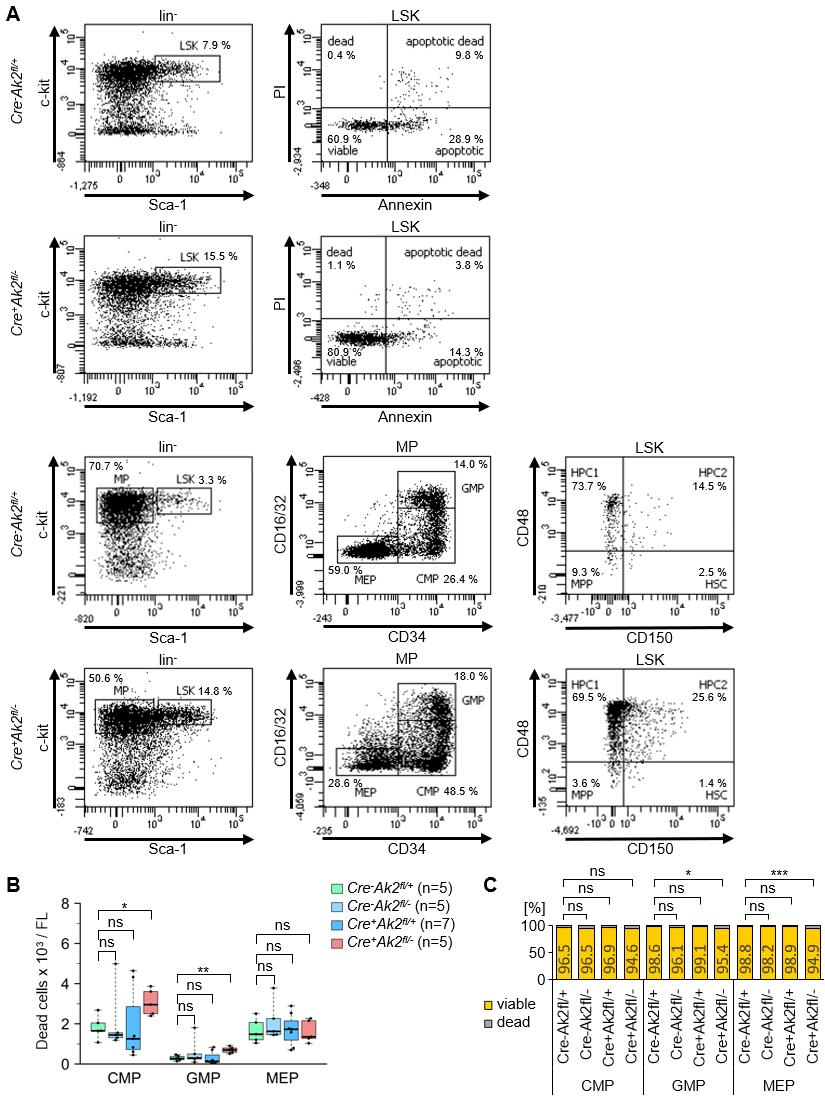


Supplemental Figure 4: Flow cytometry analyses of haematopoietic stem and progenitor cells from foetal livers (FL) of conditional E13.5 *Ak2*-knockout (*Cre^+^Ak2^fl/-^*) mice. (A) Gating strategy of LSK cells (lin^-^Sca-1^+^c-Kit^+^), myeloid progenitors (MP, lin^-^Sca-1^-^c-Kit^+^), common myeloid progenitors (CMP, lin^-^Sca-1^-^c-Kit^+^CD34^+^CD16/32^-/low^), granulocyte-macrophage progenitors (GMP, lin^-^Sca-1^-^c-Kit^+^CD34^+^CD16/32^+^), megakaryocyte-erythrocyte progenitors (MEP, lin^-^Sca-1^-^c-Kit^+^CD34^-^CD16/32^-^), haematopoietic stem cells (HSC, lin^-^Sca-1^+^c-Kit^+^CD150^+^CD48^-^), multipotent haematopoietic progenitors (MPP, lin^-^Sca-1^+^c-Kit^+^CD150^-^CD48^-^) and haematopoietic progenitor cells (HPC1 and HPC2, lin^-^Sca-1^+^c-Kit^+^CD150^-/+^CD48^+^). Representative cytometric analysis of one *Cre^-^Ak2^fl/+^* and one conditional knockout (*Cre^+^Ak2^fl/-^*) sample. Erythrocytes have been lysed and dead cells and doublets were excluded prior to analysis. Absolut number of dead (B) and relative numbers of viable and dead (C) CMP GMP and MEP cells per foetal liver. *Cre^-^Ak2^fl/+^* (n=5), *Cre^-^Ak2^fl/-^* (n=5) and *Cre^+^Ak2^fl/-^* (n=5), *Cre^+^Ak2^fl/+^* (n=7). The variance compared to *Cre^-^Ak2^fl/+^* was calculated by two-tailed Welch’s t-test, ns p > 0.05, * 0.05 ≥ p > 0.01, ** 0.01 ≥ p > 0.001 , *** p ≤ 0.001.

## Supplemental Figure 5


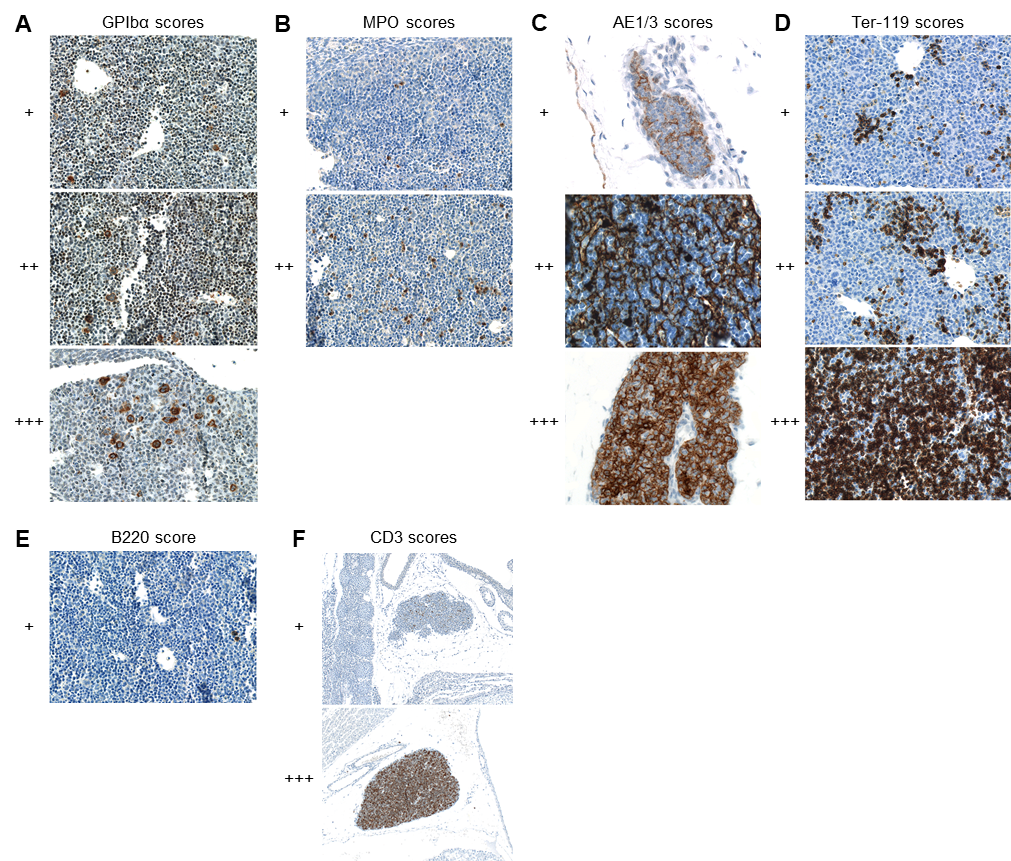


Supplemental Figure 5: Histological scores for the semiquantitative evaluation of foetal liver and thymus stainings. Representative pictures of the scores are shown for (A) GPIbα staining (foetal liver E13.5), (B) MPO staning (foetal liver (E13.5), (C) AE1/3 staining (thymus E18.5), (D) Ter-119 staining (foetal liver E13.5), (E) B220 staining (foetal liver E13.5) and CD3 staining (thymus E18.5).

## Supplemental Figure 6


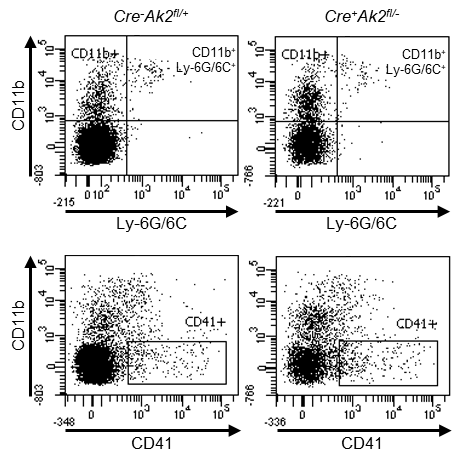


Supplemental Figure 6: Gating strategy and flow cytometric analysis of CD11b^+^ Ly-6G/6C^-^ monocytes and macrophages, CD11b^+^Ly-6G/6C^+^ granulocytes and CD41^+^ megakaryocytes in conditional E13.5 *Ak2*-knockout (*Cre^+^Ak2^fl/-^*) mice. Erythrocytes have been lysed and dead cells and doublets were excluded prior to analysis. Live/dead discrimination was done by SYTOX® Blue staining. Representative cytometric analysis of foetal liver cells of one wild type (*Cre^-^Ak2^fl/+^*) and one haematopoiesis-specific knockout embryo (*Cre^+^Ak2^fl/-^*). *Cre^-^Ak2^fl/-^ and Cre^+^Ak2^fl/+^* embryos showed no difference compared to *Cre^-^Ak2^fl/+^*.

**Supplemental Figure 7**

**
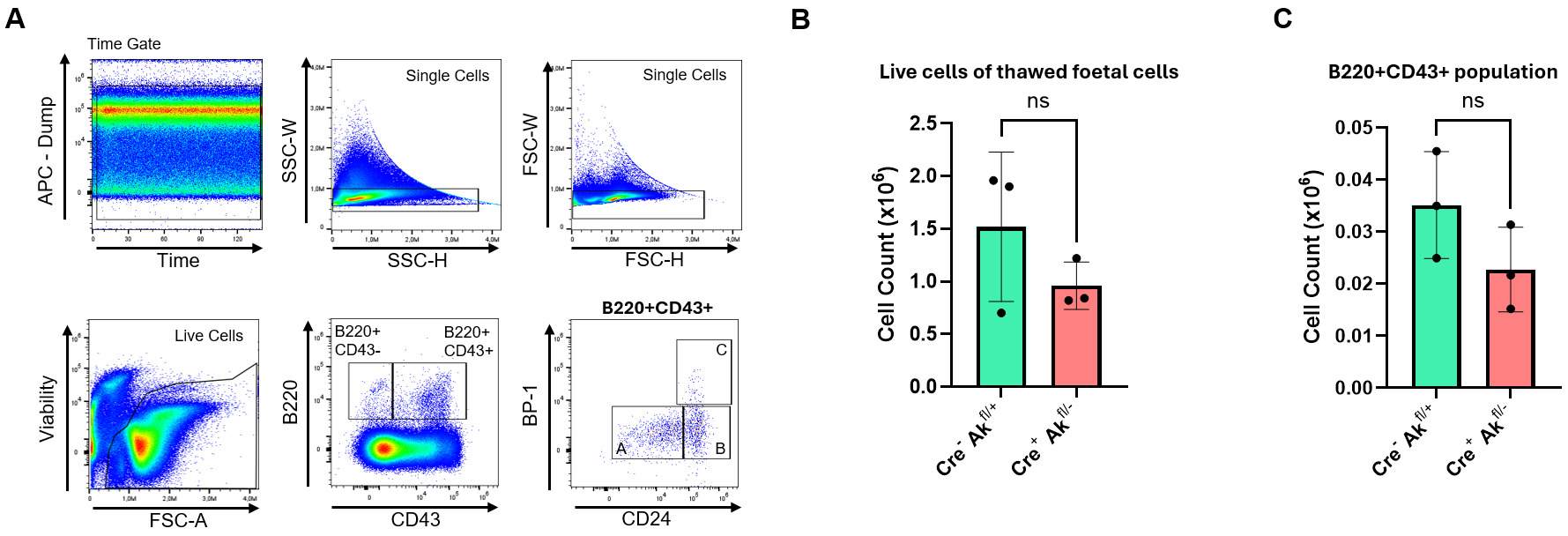
**

**
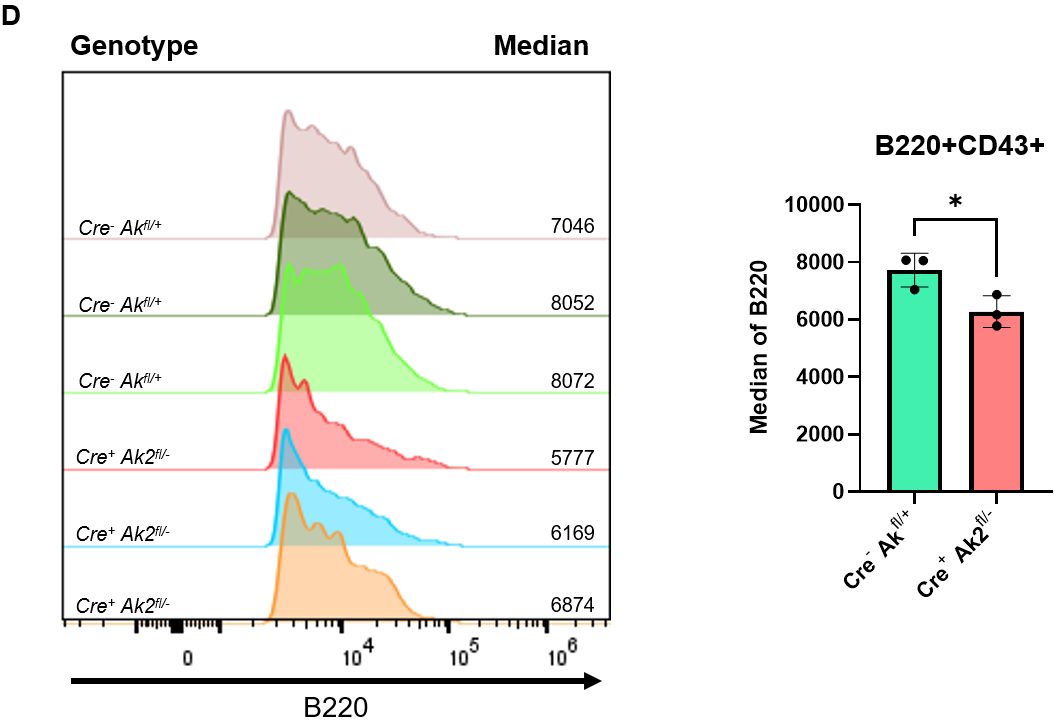
**

**Supplemental Figure 7: Flow cytometry and Vi Cell Counter analysis of foetal liver.** (A) Representative of the gating strategy for B-cell development in foetal livers using a fresh foetal liver wildtype sample (14.5 dpc). (B) Vi Cell Counter analysis of thawed foetal liver samples of wildtype (Cre^-^*Ak2^fl/+^*) and haematopoiesis-specific knockout embryo (Cre^+^*Ak2 ^fl/-^*), frozen at 13.5 dpc. (C) Total cell counts of the B220+CD43+ population of wildtype (Cre^-^*Ak2^fl/+^*) and haematopoiesis-specific knockout embryo (Cre^+^*Ak2^fl/-^*), calculated by using live cell counts of (B). (D) Histograms and statistical analysis of B220-expression levels on foetal liver B220+CD43+ cells. Statistical comparison was calculated using unpaired t-test, ns p > 0.05, * 0.05 ≥ p > 0.01.

## Supplemental Figure 8


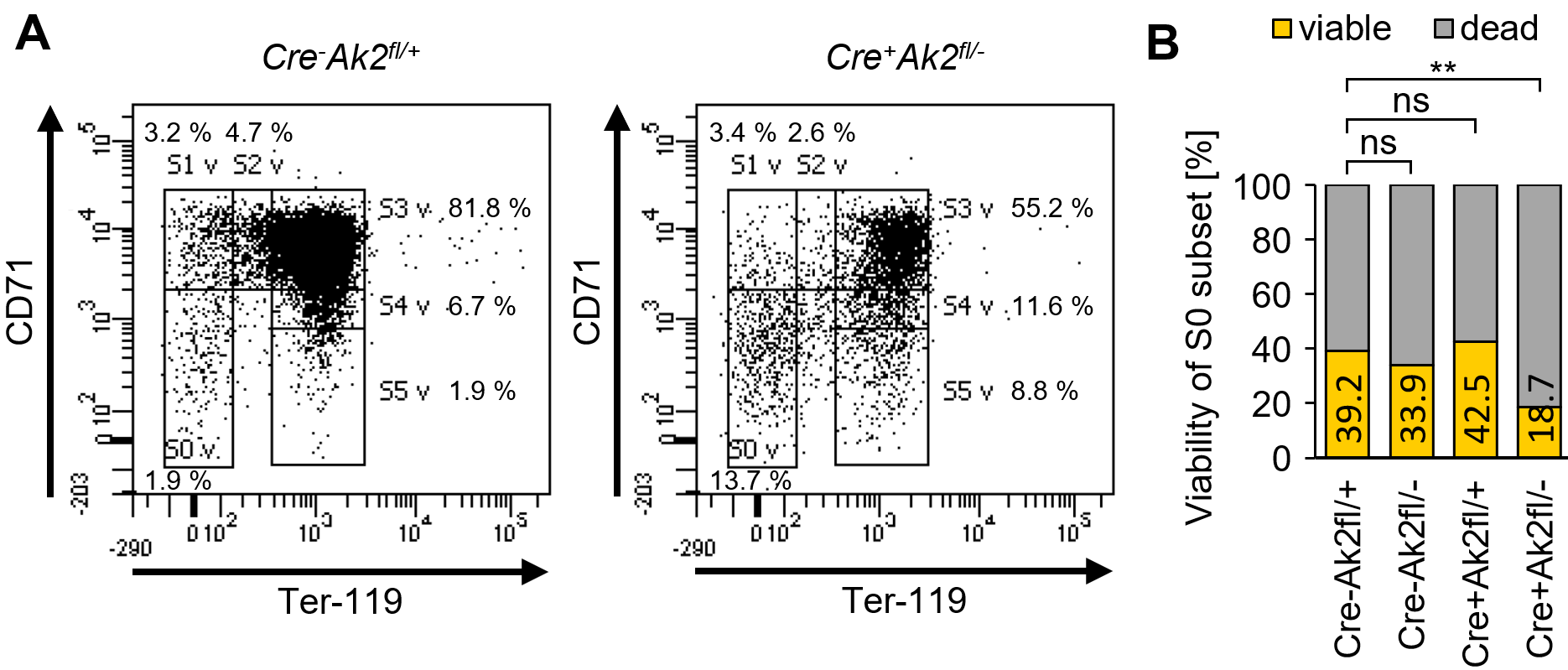


Supplemental Figure 8: Flow cytometry analyses of erythroid development in **E13.5 *Cre^+^Ak2^fl/-^*** foetal livers. (A) Gating strategy and flow cytometric analyses of erythroid progenitor cells. Viable cells were separated into six subsets (S0 – S5) according to their Ter-119 and CD71 expressions in the absence of the lineage markers B220, CD3e, CD11b, CD41 and Gr-1. Live/dead discrimination was done by SYTOX® Blue staining. Representative cytometric analysis of one *Cre^-^Ak2^fl/+^* and one *Cre^+^Ak2^fl/-^* sample. *Cre^-^Ak2^fl/-^ and Cre^+^Ak2^fl/+^* embryos showed no difference compared to *Cre^-^Ak2^fl/+^*. (B) Relative numbers of viable and dead S0 cells per foetal liver. *Cre^-^Ak2^fl/+^* (n=6), *Cre^-^Ak2^fl/-^* (n=8), *Cre^+^Ak2^fl/+^* (n=7) and *Cre^+^Ak2^fl/-^* (n=8). The variance compared to *Cre^-^Ak2^fl/+^* was calculated by two-tailed Welch’s t-test, ns p > 0.05, ** p ≤ 0.01.

## Supplemental Figure 9

**min max**

Supplemental Figure 9: Differential mRNA expression of selected kinases during erythropoiesis. Human and murine mRNA expression of kinases as compiled in the BloodSpot database (www.bloodspot.eu). Expression values are normalized, summarized measured probe intensities (Robust Multi-array Average (RMA)), which have further been batch corrected. The minimal and maximal values of the color code are the minimal and maximal reported value for each kinase in the shown human and murine haematopoietic cell types according to the bloodspot database. *AK1*, *AK2* and *AK4*: adenylate kinases 1, 2 and 4; *CKMT2:* mitochondrial creatine kinase 2; CKM: M-type creatine kinase; LT-HSC: long term hematopoietic stem cell; ST-HSC: short term hematopoietic stem cell; CMP: common myeloid progenitor; MEP: megakaryocyte/erythroid progenitor; ProE: erythroid progenitor cell, nucl. Erythrocytes: nucleated erythrocytes, na: data not available.

## Supplemental Figure 10


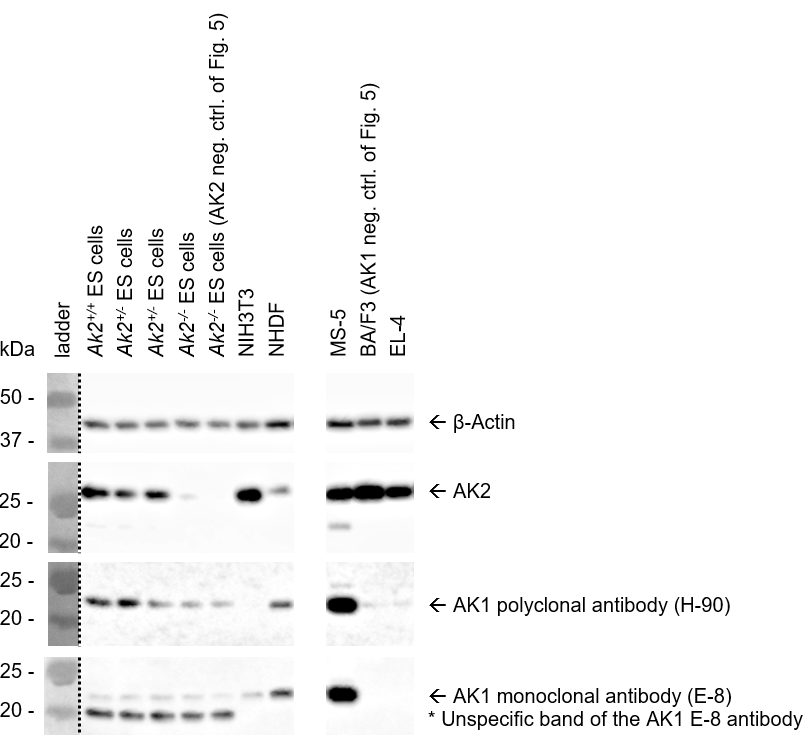


Supplemental Figure 10: Comparison of the polyclonal AK1 antibody (H-90) and the monoclonal AK1 antibody (E-8), which was used in this study. Western Blot analysis of 10 µg protein lysate per lane of murine ES cells, the murine cell lines NIH3T3, MS-5, BA/F3 and EL-4 and neonatal human dermal fibroblasts (NHDF). Imaging mode: 1 min 3x3 binning (β-Actin and AK2), 30 min 3x3 binning (AK1 H-90) and 5 min 3x3 binning (AK1 E-8) with the Fusion SL imaging system (Vilber Lourmat). Dashed lines: pre-stained protein standard ladders were documented separately to chemiluminescence detection.

## Supplemental Table 1

Supplemental Table 1: Genotype distribution of offspring from *Ak2^+/-^* x *Ak2^+/-^* matings. Genotyping of live-born mice and embryos from *Ak2^+/-^* x *Ak2^+/-^* matings revealed a wild type to heterozygous ratio of 1:2 and a loss of *Ak2*-knockout mice from E10.5 to postnatal mice. For embryos at E8.5 and embryonic blastocyst cells at E3.5, all genotypes were obtained in normal Mendelian ratios. The ratio of wild type (*Ak2^+/+^*) to heterozygous (*Ak2^+/-^*) animals or the ratio of wild-type to heterozygous and knockout animals was analyzed for significant deviation from the expected 1:2 or 1:2:1 ratio by Chi-square test for goodness of fit. *Cumulative binomial probability of obtaining the number of *Ak2^-/-^* animals or less under the null hypothesis. Values of p ≤ 0.001 are shaded in red. *NA* indicates not applicable.

## Supplemental Table 2

Supplemental Table 2: Genotype distribution of offspring from *Ak2^fl/fl^* x *vav-iCre AK2^+/-^* matings. A reduced frequency of conditional *Ak2*-knockout (*Cre^+^Ak2^fl/-^*) embryos was detected at E18.5 and no live-born conditional *Ak2*-knockout could be detected. The ratio of all four possible genotypes to each other was analyzed for significant deviation from the expected 1:1:1:0 or 1:1:1:1 ratio by Chi-square test for goodness of fit. *The significance of a deficiency of conditional *Ak2*-knockout animals was evaluated by calculating the cumulative binomial probability of obtaining the indicated number of *Cre^+^Ak2^fl/-^* animals or less under the null hypothesis. Significant values of p ≤ 0.05 are shaded in red. *NA*: not applicable.

## Supplemental Table 3

Supplemental Table 3: Antibodies used for flow cytometric analyses. LSK: lineage^-^ Sca-1^+^ c-Kit^+^, APC: allophycocyanin, FITC: fluorescein, PE: phycoerythrin, Cy7: cyanine 7, AF: Alexa Fluor, BV: Brilliant Violet.

| **Staining** | **Dilution factor** | **Antibody** | **Coupled Dye** | **Manufacturer** | **Cat. #** | **Lot #** | **Isotype** |
| --- | --- | --- | --- | --- | --- | --- | --- |
| **Blocking solution** | 1:20 | Mouse IgG | none | Jackson | 015-000-003 | 128233 | Mouse IgG |
|  | 1:100/200 | Anti-CD16/CD32 | none | BD | 553142 | 3135956 | Rat IgG2b, κ |
| **LSK cells** | 1:800 | Anti-CD4 | Biotin | BD | 553728 | 4010712 | Rat LEW IgG2b, κ |
|  | 1:500 | Anti-Ly-6G/6C | Biotin | eBioscience | 13-5931-85 | E03075-1632 | Rat IgG2b, κ |
|  | 1:400 | Anti-Ter-119 | Biotin | eBioscience | 13-5921-85 | E03071-1632 | Rat IgG2b, κ |
|  | 1:200 | Anti-CD8a | Biotin | eBioscience | 13-0081-85 | E02386-1633 | Rat IgG2a, κ |
|  | 1:200 | Anti-B220 | Biotin | BD | 553086 | 2279903 | Rat IgG2a, κ |
|  | 1:100 | Anti-CD3e | Biotin | BD | 553239 | 5202690 | Hamster IgG2, κ |
|  | 1:100 | Anti-CD5 | Biotin | eBioscience | 13-0051 | E02374-1632 | Rat IgG2a, kappa |
|  | 1:200 | Anti-c-Kit | APC | BD | 553356 | 4084660 | Rat WI IgG2b, κ |
|  | 1:100 | Anti-Sca-1 | PE-Cy7 | BD | 558162 | 5329775 | Rat LEW IgG2a, κ |
| **Haematopoietic stem cells** | LSK cell staining + | | | | | | |
|  | 1:100 | Anti-CD150 | PE | eBioscience | 12-1502-80 | E09989-1634 | Rat IgG2b |
|  | 1:100 | Anti-CD48 | FITC | eBioscience | 11-0481-82 | E021043 | Hamster IgG |
| **Haematopoietic progenitor cells** | LSK cell staining + | | | | | | |
|  | 1:100 | Anti-CD16/32 | PE | eBioscience | 12-0161-81 | 4274519 | Rat IgG2a, λ |
|  | 1:25 | Anti-CD34 | FITC | eBioscience | 11-0341-85 | E00265-1633 | Rat IgG2a, κ |
| **Erythroid progenitors** | 1:800 | Anti-CD11b | PE | eBioscience | 12-0112-82 | E01073-207 | Rat IgG2b, k |
|  | 1:400 | Anti-CD41 | PE | BD | 558040 | 4141653 | Rat IgG1, κ |
|  | 1:400 | Anti-B220 | PE | BD | 553090 | 6701 | Rat IgG2a, κ |
|  | 1:400 | Anti-Ly-6G/6C | PE | BD | 553128 | 75542 | Rat IgG2b, κ |
|  | 1:25 | Anti-CD3e | PE | BD | 555275 | 3277640 | Rat SD IgG2b, κ |
|  | 1:100 | Anti-CD71 | FITC | BD | 561936 | 5243811 | Rat WF IgG1, κ |
|  | 1:100 | Anti-Ter-119 | APC | BD | 557909 | 42622 | Rat WI IgG2b, κ |
| **Myeloid cells** | 1:800 | Anti-CD11b | PE-Cy7 | eBioscience | 25-0112-82 | E07514-1249 | Rat IgG2b, κ |
|  | 1:400 | Anti-CD41 | PE | BD | 558040 | 4141653 | Rat IgG1, κ |
|  | 1:200 | Anti-Ly-6G/6C | FITC | BD | 553126 | 26563 | Rat IgG2b, κ |
| **B-cell colony assay control** | 1:200 | Anti-CD19 | PE | BD | 553786 | 72092 | Rat IgG2a, κ |
|  | 1:100 | Anti-B220 | APC | BD | 553092 | 73008 | Rat IgG2a, κ |
| **B-cell panel frozen foetal liver** | 1:100 | Anti-CD19 | AF 700 | eBioscience | 56-0193-82 | E08967-1630 | Rat IgG2a, κ |
|  | 1:200 | Anti-B220 | PE Fire810 | Biolegend | 103287 | B363716 | Rat IgG2a, κ |
|  | 1:200 | Anti-IgM | PerCP eF710 | eBioscience | 46-5790-82 | E10793-101 | Rat IgG2a, κ |
|  | 1:400 | Anti-IgD | Spark NIR685 | Biolegend | 405749 | B384585 | Rat IgG2a, κ |
|  | 1:400 | Anti-CD43 | PE Dazzle 594 | Biolegend | 121226 | B237204 | Rat IgG2b, κ |
|  | 1:1000 | Anti-CD24 | Pacific Blue | Biolegend | 101820 | B271348 | Rat IgG2b, κ |
|  | 1:50 | Anti-BP-1 | PE | eBioscience | 12-5891-82 | 2473669 | Rat IgG2a, κ |
|  | 1:100 | Anti-IL7-Ra | PE-Cy7 | Biolegend | 135014 | B164275 | Rat IgG2a, κ |
|  | 1:200 | Anti-Ter-119 | APC | Biolegend | 116212 | B185639 | Rat IgG2b, κ |
|  | 1:3200 | Anti-CD4 | APC | BD | 553051 | 1113645 | Rat IgG2a, κ |
|  | 1:400 | Anti-CD8a | APC | eBioscience | 17-0081-82 | 2023410 | Rat IgG2a, κ |
|  | 1:200 | Anti-Ly6G | APC | BD | 127614 | B366717 | Rat IgG2a, κ |
|  | 1:100 | Anti-NK1.1 | APC | BD | 550627 | 5254819 | Rat IgG2a, κ |
|  | 1:1600 | Anti-CD11b | APC | Biolegend | 101212 | B279418 | Rat IgG2b, κ |
|  | 1:100 | Anti-CD93 | Biotin | eBioscience | 13-5892-85 | 2553013 | Rat IgG2b, κ |
|  | 1:300 | Anti-Streptavidin | BV605 | Biolegend | 405229 | B422127 | none |
|  | 1:200 | Anti-Ly6D | FITC | Biolegend | 138606 | B166784 | Rat IgG2c, κ |
|  | 1:1000 | Viability | Zombie Aqua | Biolegend | 423101 | none | None |

## Supplemental Table 4

Supplemental Table 4: Cell populations sorted for digital PCR and Western Blot analyses. The different cell populations were identified according to their surface marker expression as indicated. a) lin: B220, CD3e, CD4, CD5, CD8a, Ly-6G/6C and Ter-119, b) lin: B220, CD3e, CD41 and Ly-6G/6C.

| **Cell type** | **Marker** | **Source** |
| --- | --- | --- |
| LSK cells | lin^- a)^ Sca-1^+^ c-Kit^+^ | Foetal liver |
| Murine embryonic fibroblasts | CD45^+^ | Cell culture |
| Granulocytes | CD11b^+^ Ly-6G/6C^+^ | Foetal liver |
| Megakaryocyte | CD41^+^ | Foetal liver |
| Erythroid progenitors S0 | lin^- b)^ CD71^-^ Ter-119^-^ | Foetal liver |
| Erythroid progenitors S3 | lin^- b)^ CD71^+^ Ter-119^+^ | Foetal liver |
| Human erythrocytes (adult) | Ter-119^+^ | Blood |
| Murine erythrocytes (adult) | Ter-119^+^ | Blood |
| Murine erythrocytes (foetal) | lin^- b)^ Ter-119^+^ | Foetal liver |

# Supplemental methods

## Genotyping of mice

The KAPA Mouse Genotyping Hot Start Kit from peqlab (cat. # 07-KK7352) was used according to the manufacturer’s instructions with a reduced extraction buffer volume of 50 µl per sample and a reduced PCR reaction volume of 20 µl. The three primers Oligo1 (5'-TGCAGAAGACAAGGTAGAGC-3'), Oligo2 (5'-TGAAGCCTATGTGTGGAA

GG-3') and Oligo3 (5'-TCTCACCCATGCTTTCGTC-3') were used to identify wild-type (260 bp), floxed (379 bp) and knockout alleles (427 bp). The primers vm57hCre (5'-CTCCAACCTGCTGACTGTGCACCAA-3') and vm58hCre (5'-CCATCTCTCCACCAG

CTTGGTAAC-3') were used for vav-iCre detection (670 bp).

## Isolation of foetal liver cells

Foetal livers were transfered to Dulbecco's phosphate-buffered saline (PBS) (Gibco, #14190094) with 5% foetal calf serum (FCS) (GE Healthcare, CH30160.03) and the cell suspension was filtered through a nylon mesh (Sefar, # 3A03-0150-102-00) to obtain a debris-free single-cell solution. The number of viable cells was determined by using a hemocytometer and Trypan Blue staining (Gibco, #15250061).Prior to flow cytometric analyses of foetal liver cells, red blood cells were lysed with erythrocyte lysis buffer (155 mM ammonium chloride, 10 mM potassium bicarbonate, 100 µM ethylenediaminetetraacetic acid (EDTA), pH 7.2-7.4) except for erythroid progenitor stainings. The number of viable cells after red blood cell lysis was determined with a haemocytometer. Single staining and fluorescence minus one control containing an isotype control were used for each staining. The samples were analysed with a FACSCanto II Flow Cytometer (BD Biosciences). The lineage^-^ Sca-1^+^ c-Kit^+^ cell population was sorted using a FACSAria II (BD Biosciences) cell sorter, all other cell populations were sorted using a FACSAria (BD Biosciences) cell sorter.

**Flow Cytometry analyses**

One million foetal liver cells per sample were used directly or after red blood cell lysis. Unspecific antibody-binding was blocked with blocking solution (1:20 Mouse IgG, Jackson, #015-000-003; 1:100 Anti-CD16/CD32, BD, #553142) followed by the specific staining. All antibodies used for the different stainings and the dilution factors are listed in Supplemental Table 3. When biotin-labeled antibodies were used, the staining procedure was repeated with Qdot 605 streptavidin conjugate (Invitrogen, cat. # Q10001MP, lot # 1743120, diluted 1:400). After the staining, the cells were resuspended in 200 μl FACS buffer containing the viability dye SYTOX® Blue (SYTOX® Blue Dead Cell stain in PBS + 5% FCS, 1:20.000). When appropiate, all flow cytometry analyses were carried out in accordance with the guidelines published by Cossarizza et al. [2].

**Flow Cytometry analyses of frozen foetal liver cells**

Frozen foetal liver samples were thawed in 37°C water bath and washed twice in 15 ml FACS wash (10% 10xPBS, 2% FBS, 0.4% 0.5 M EDTA, diluted in deionized water). Cells were counted and viability was calculated on a Vi-Cell Counter (Beckman Coulter). Equal numbers of foetal liver cells per sample were plated into 96-well plate and stained for viability at room temperature for 20 min using Zombie Aqua or ViaDye Red in PBS. After washing, unspecific antibody-binding was blocked with blocking Ab (1:200 Anti-CD16/32) in combination with staining for biotin (30 min, 4°C), followed by specific staining (30 min, 4°C). All antibodies used for the different stains and the dilution factors are listed in Supplemental Table 3. All staining steps were performed in 50µl volume using FACS wash. After the staining, the cells were resuspended in 180 µl FACS wash and analyzed on a 3 Laser Cytek Northern Lights flow cytometer. Gating strategy was chosen based on Hardy et al. [3].

**Fluorescence-activated cell sorting**

Fluorescence-activated cell sorting was used to isolate the cell types listed in Supplemental Table 4 for digital PCR or Western Blot analyses from the indicated source.

## Digital PCR

The QuantStudio 3D Digital PCR Master Mix v2 kit (ThermoFisher Scientific, #A26358) was used following the manufacturer’s instructions. Genomic DNA (12 ng/µl) or lysates of 1000 sorted cells per µl (using the KAPA Mouse Genotyping Hot Start Kit, peqlab, #07-KK735) were used as template. The reference exon (*Ak2* exon 2) was detected with a VIC-labelled TaqMan^®^ Assay (ThermoFisher Scientific, #4400291, Assay-ID: Mm00112530_cn, custom design with VIC instead of FAM), while a FAM-labelled TaqMan^®^ Assay (ThermoFisher Scientific, #4400291, Assay-ID: Mm00112533_cn) was used to analyze the presence or absence of the floxed *Ak2* exon 3. The quality cut off for quantification was set at >300 positive wells per 20K chip for reference exon 2.

**Western Blots**

Whole-protein lysates were separated on a 15% polyacrylamide gel by SDS-PAGE and transferred to polyvinylidene fluoride membranes (PVDF membrane, Millipore, #IPVH00010) following the manufacturer’s instructions with a semi-dry transfer unit (TE77ECL, Amersham Biosciences). For immunodetection the dried membrane was blocked with 5% skimmed milk powder in Tris-buffered saline with Tween. The following antibodies were used: anti-AK1 (1:1,000, Santa Cruz Biotechnology, #sc-365316), anti-AK2 (1:20,000, Proteintech Europe, #11014-1-AP), anti-GAPDH (1:40,000, Abcam, #ab181602), anti-rabbit IgG (1:3,000, (H+L)-HRP conjugate, BioRad, #170-6516), anti-mouse IgG (1:3,000, (H+L)-HRP conjugate, BioRad, #170-6515). Horseradish peroxidase labelled secondary antibodies were detected by a chemiluminescent substrate (SuperSignal® West Pico Chemiluminescent Substrate, Thermo Scientific, cat. # 34080) following the manufacturer’s instructions.

# Supplemental results

## A constitutive *Ak2*-knockout causes early embryonic lethality in mice

To mimic human RD, heterozygous mice with an *Ak2* exon 3-4 deletion of one allele (*Ak2^+/-^* mice) were inter-crossed. Genotyping did not detect homozygous *Ak2*-knockout mice from embryonic day 10.5 (E10.5) to postnatal mice (Supplemental Table 1). No *Ak2^-/-^* resorption bodies were detectable at E10.5 or later. Wild type and heterozygous embryos developed normally (1) (Supplemental Figure 2). Homozygous *Ak2*-knockout embryos at E8.5 were growth retarded and their morphology corresponded to an expected morphology at E6.25-7.25 [1].

**Supplemental references**

1. **Theiler K**. *The House Mouse: Atlas of Embryonic Development*. Berlin, Heidelberg: Springer Berlin Heidelberg; 1989. Available at: http://link.springer.com/10.1007/978-3-642-88418-4 [Accessed March 4, 2025].DOI: 10.1007/978-3-642-88418-4.

2. **Cossarizza A, Chang H, Radbruch A, Abrignani S, Addo R, Akdis M, Andrä I, *et al.*** Guidelines for the use of flow cytometry and cell sorting in immunological studies (third edition). *Eur. J. Immunol.* 2021; **51**:2708–3145. DOI: 10.1002/eji.202170126.

3. **Hardy RR**, **Li YS**, **Allman D**, **Asano M**, **Gui M**, **Hayakawa K**. B-cell commitment, development and selection. *Immunol. Rev.* 2000; **175**:23–32. DOI: 10.1111/j.1600-065X.2000.imr017517.x.

Western Blot raw data

## Unprocessed original images of Figure 5A (upper panel)


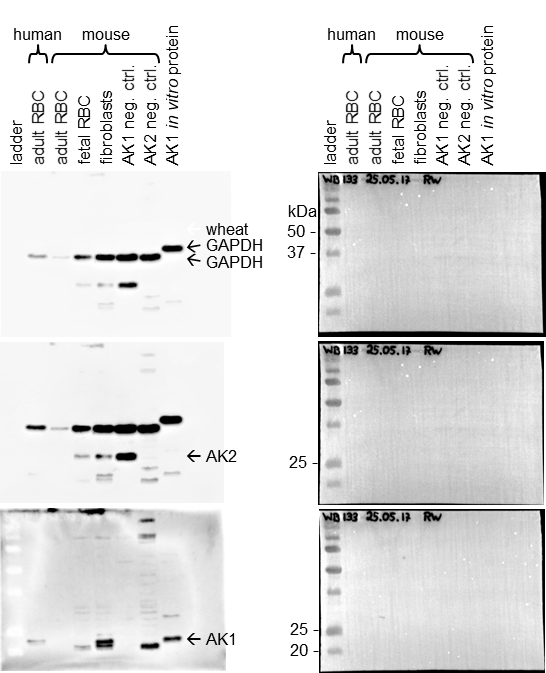


Protein expression of AK1 and AK2 in human and murine erythrocytes. Western Blot analysis of 8 µg protein. Red boxes indicate the image details used in Figure 5A. Imaging mode: 1 min 3x3 binning (AK1 and AK2) and 30 sec 3x3 binning (GAPDH) with the Fusion SL imaging system (Vilber Lourmat). Loading control: mammalian and wheat GAPDH, AK1 neg. ctrl.: BA/F3 cell line, AK2 neg. ctrl.: *Ak2*^-/-^ murine ES cells

## Unprocessed original images of Figure 5A (lower panel)


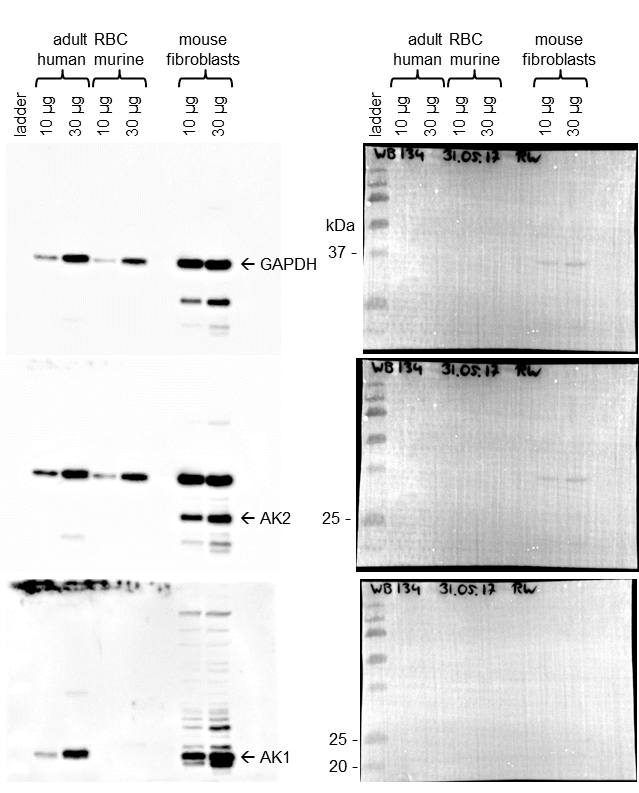


Protein expression of AK1 and AK2 in human and murine erythrocytes. Western Blot analysis of 10 µg and 30 µg protein per lane. Red boxes indicate the image details used in Figure 5A. Imaging mode: 1 min 3x3 binning (AK1 and AK2) and 30 sec 3x3 binning (GAPDH) with the Fusion SL imaging system (Vilber Lourmat). Loading control: mammalian and wheat GAPDH, AK1 neg. ctrl.: BA/F3 cell line, AK2 neg. ctrl.: *Ak2*^-/-^ murine ES cells.

## Unprocessed original images of Supplemental Figure 10


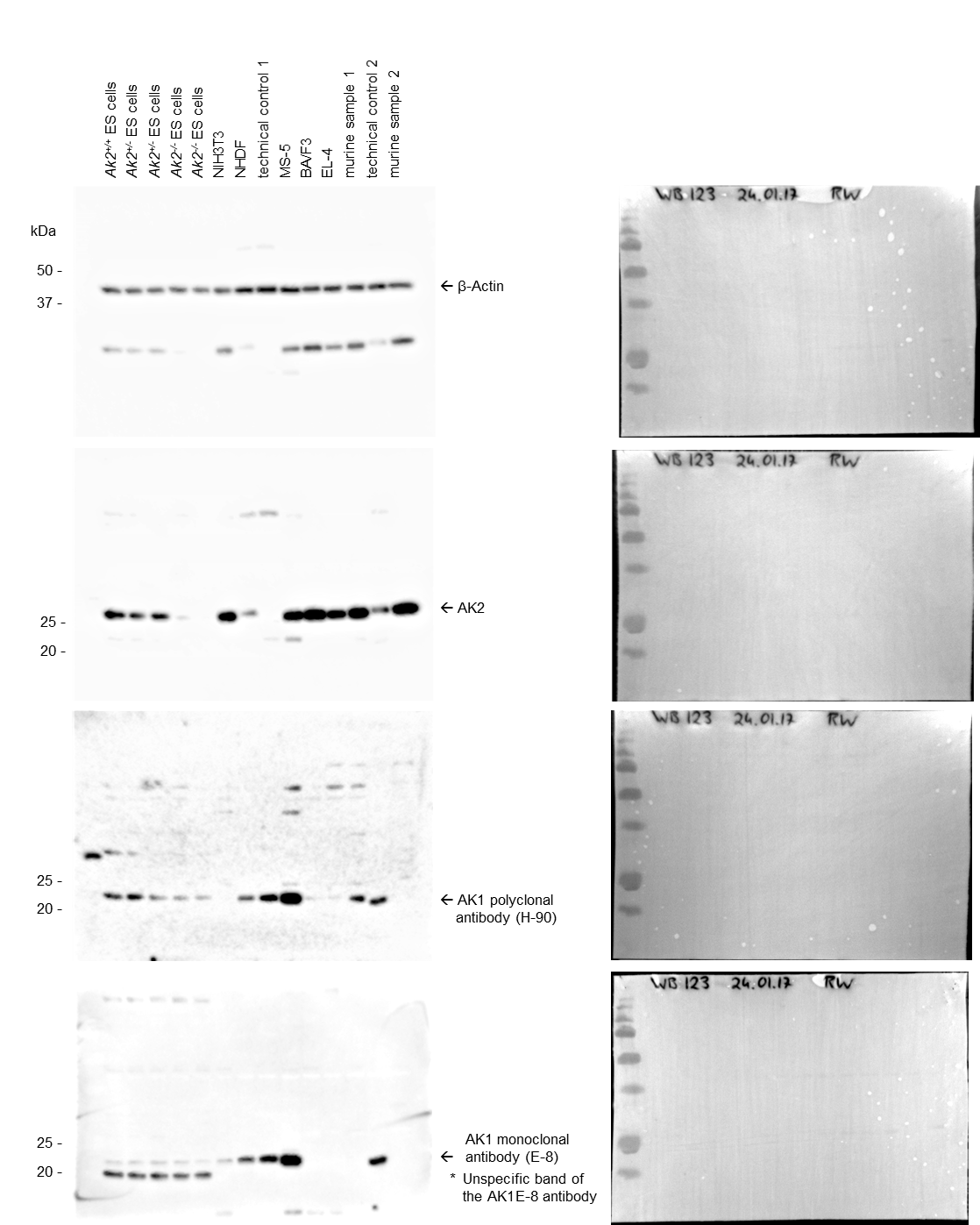


Western Blot analysis of 10 µg protein lysate per lane of murine ES cells, the murine cell lines NIH3T3, MS-5, BA/F3 and EL-4 and neonatal human dermal fibroblasts (NHDF). Red boxes indicate the image details used in Supplemental igure 10. Imaging mode: 1 min 3x3 binning (β-Actin and AK2), 30 min 3x3 binning (AK1 H-90) and 5 min 3x3 binning (AK1 E-8) with the Fusion SL imaging system (Vilber Lourmat).
